# Peace Lessons from an Unlikely Source

**DOI:** 10.1371/journal.pbio.0020101

**Published:** 2004-04-13

**Authors:** Frans B. M de Waal

## Abstract

How much is the aggression we observe in nonhuman primates the result of culture, and will the answer provide insights into our own violent behaviour?

Upon arrival from Europe, now more than two decades ago, I was taken aback by the level of violence in the American media. I do not just mean the daily news, even though it is hard getting used to multiple murders per day in any large city. No, I mean sitcoms, comedies, drama series, and movies. Staying away from Schwarzenegger and Stallone does not do it; almost any American movie features violence. Inevitably, desensitization sets in. If you say, for example, that *Dances with Wolves* (the 1990 movie with Kevin Costner) is violent, people look at you as if you are crazy. They see an idyllic, sentimental movie, with beautiful landscapes, showing a rare white man who respects American Indians. The bloody scenes barely register.

Comedy is no different. I love, for example, *Saturday Night Live* for its inside commentary on peculiarly American phenomena, such as cheerleaders, televangelists, and celebrity lawyers. But *SNL* is incomplete without at least one sketch in which someone's car explodes or head gets blown off. Characters such as Hans and Franz (“We're going to pump you up!”) appeal to me for their names alone (and yes, I do have a brother named Hans), but when their free weights are so heavy that their arms get torn off, I am baffled. The spouting blood gets a big laugh from the audience, but I fail to see the humor.

Did I grow up in a land of sissies? Perhaps, but I am not mentioning this to decide whether violence in the media and our ability to grow immune to it—as I also have over the years—is desirable, or not. I simply wish to draw attention to the cultural fissures in how violence is portrayed, how we teach conflict resolution, and whether harmony is valued over competitiveness. This is the problem with the human species. Somewhere in all of this resides a human nature, but it is molded and stretched into so many different directions that it is hard to say if we are naturally competitive or naturally community-builders. In fact, we are both, but each society reaches its own balance between the two. In America, the squeaky wheel gets the grease. In Japan, the nail that stands out gets pounded into the ground.

Does this variability mean, as some have argued, that animal studies cannot possibly shed light on human aggression? “Nature, red in tooth and claw” remains the dominant image of the animal world. Animals just fight, and that is it? It is not that simple. First, each species has its own way of handling conflict, with for example the chimpanzee (Pan troglodytes) being far more violent than that equally close relative of ours, the bonobo (P. paniscus) (de Waal 1997). But also within each species we find, just as in humans, variation from group to group. There are “cultures” of violence and “cultures” of peace. The latter are made possible by the universal primate ability to settle disputes and iron out differences.

There was a time when no review of human nature would be complete without assertions about our inborn aggressiveness. The first scientist to bring up this issue, not coincidentally after World War II, was Konrad Lorenz (1966). Lorenz's thesis was greeted with accusations about attempts to whitewash human atrocities, all the more so given the Nobel Prize winner's native tongue, which was German. But Lorenz was hardly alone. In the USA, science journalist Robert Ardrey (1961) presented us as “killer apes” unlikely to ever get our nasty side under control. Recent world events have done little to counter this pessimistic outlook.

The opposition argued, of course, that aggression, like all human behavior, is subject to powerful cultural influences. They even signed petitions to this effect, such as the controversial *Seville Statement on Violence* (Adams et al. 1990). In the polarized mind-set of the time, the issue was presented in either-or fashion, as if behavior cannot be both learned and built upon a biological foundation. This rather fruitless nature/nurture debate becomes considerably more complex if we include what is usually left out, which is the ability to keep aggression under control and foster peace. For this ability, too, there exist animal parallels, such as the habit of chimpanzees to reconcile after fights by means of a kiss and embrace. Such reunions are well-documented in a multitude of animals, including nonprimates, such as hyenas and dolphins. They serve to restore social relationships disturbed by aggression, and any animal that depends on cooperation needs such mechanisms of social repair (Aureli and de Waal 2000; de Waal 2000). There are even indications that in animals, too, cultural influences matter in this regard. This may disturb those who write culture with a capital *C*, and hence view it as uniquely human, but it is a serious possibility nonetheless.

Nonhuman culture is currently one of the hottest areas in the study of animal behavior. The idea goes back to the pioneering work of Kinji Imanishi, who in 1952 proposed that if individuals learn from one another, their behavior may over time grow different from that of individuals in other groups of the same species, thus creating a characteristic culture (reviewed by de Waal 2001). Imanishi thus brought the culture concept down to its most basic feature, that is, the social rather than genetic transmission of behavior. Since then, many examples have been documented, mostly concerning subsistence techniques, such as the sweet potato washing of Japanese macaques (Macaca fuscata) and the rich array of tool use by wild chimpanzees, orangutans (Pongo pymaeus), and capuchin monkeys (Cebus spp.) (Whiten et al. 1999; de Waal 2001; Hirata et al. 2001; Perry et al. 2003; van Schaik et al. 2003). However, much less attention has been paid to *social culture*, which we might define as the transmission of social positions, preferences, habits, and attitudes.

Social culture is obviously harder to document than tool use. In human culture, for instance, it is easy to tell if people eat with knife and fork or with chopsticks, but to notice if a culture is egalitarian or hierarchical, warm or distant, collectivistic or individualistic takes time and is difficult to capture in behavioral measures. A well-documented monkey example of social culture is the inheritance of rank positions in macaque and baboon societies. The future position in the hierarchy of a newborn female can be predicted with almost one hundred percent certainty on the basis of her mother's rank. Females with relatives in high places are born with a silver spoon in their mouth, so to speak, whereas those of lowly origin will spend their life at the bottom. Despite its stability, the system depends on learning. Early in life, the young monkey finds out against which opponents it can expect help from her mother and sisters. When sparring with peer A she may utter screams that recruit massive support to defeat A. But against peer B she can scream her lungs out and nothing happens. Consequently, she will come to dominate A but not B. Experiments manipulating the presence of family members have found that when support dwindles dominant females are unable to maintain their positions (Chapais 1988). In other words, the kin-based hierarchy is maintained for generation after generation through social rather than genetic transmission.

Returning to the issue of aggressive behavior, here the effects of social culture can be felt as well. Without any drugs or brain lesions, one experiment managed to turn monkeys into pacifists. Juveniles of two different macaque species were placed together, day and night, for five months. Rhesus monkeys (Macaca mulatta), known as quarrelsome and violent, were housed with the more tolerant and easy-going stumptail monkeys (M. arctoides) (Figure 1). Stumptail monkeys easily reconcile with their opponents after fights by holding each others' hips (the so-called “hold-bottom” ritual), whereas reconciliations are rare in rhesus monkeys. Because the mixed-species groups were dominated by the stumptails, physical aggression was rare. The atmosphere was relaxed, and after a while all of the monkeys became friends. Juveniles of the two species played together, groomed together, and slept in large, mixed huddles. Most importantly, the rhesus monkeys developed peacemaking skills on a par with those of their more tolerant group mates. Even when, at the end of the experiment, both species were separated, the rhesus monkeys still showed three times more reconciliation and grooming behaviors after fights than typical of their kind (de Waal and Johanowicz 1993). Primates thus can adopt social behavior under the influence of others, which opens the door to social culture.

**Figure 1:**
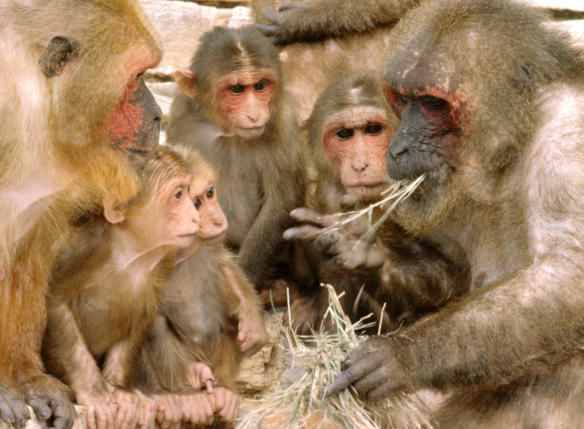
Stumptail Monkeys Stumptail monkeys (Macaca arctoides) are among the most conciliatory members of the genus Macaca. They are heavily built, yet remarkably friendly and tolerant, such as here: the alpha male is eating attractive food unperturbed by an entire audience around him. When stumptail monkeys were housed with a less tolerant macaque, they modified the latter species' behavior into a more pacific direction. (Photograph by Frans de Waal, used with permission.)

Not unlike rhesus monkeys, baboons have a reputation for fierce competition and nasty fights. With the study by Robert Sapolsky and Lisa Share published in this issue of *PLoS Biology*, we now have the first field evidence that primates can go the flower power route (Sapolsky and Share 2004). Wild baboons developed an exceptionally pacific social tradition that outlasted the individuals who established it. For years, Sapolsky has documented how olive baboons (Papio anubis) on the plains of the Masai Mara, in Kenya, wage wars of nerves, compromising their rivals' immune systems and pushing up the level of their blood cortisol (Sapolsky 1994). An accident of history, however, selectively wiped out all the male bullies of his main study troop. As a result, the number of aggressive incidents dropped dramatically. This by itself was not so surprising. It became more interesting when it was discovered that the behavioral change was maintained for a decade. Baboon males migrate after puberty, hence fresh young males enter troops all the time, resulting in a complete turn-over of males during the intervening decade. Nevertheless, compared with troops around it, the affected troop upheld its reduced aggression, increased friendly behavior, and exceptionally low stress levels. The conclusion from this natural experiment is that, like human societies, each animal society has its own ecological and behavioral history, which determines its prevalent social style.

It is somewhat ironic that at a time when researchers on human aggression are increasingly attracted, albeit with a far more sophisticated approach, to the Lorenzian idea of a biological basis of aggression (Enserink 2000), students of animal behavior are beginning to look at its possible cultural basis. There is no reason for animals with a development as slow as a baboon (with adulthood achieved in five or six years) not to be influenced in every way by the environment in which they grow up, including the social environment. How this influence takes place is a point of much debate, and remains unclear in the case of the peaceful male baboons in the Masai Mara. Given their mobility, the males themselves are unlikely transmitters of social traditions within their natal troop. Therefore, Sapolsky and Share look at the females for an answer—female baboons stay all their lives in the same troop. By reacting positively to certain kinds of behavior, for example, females may be able to steer male attitudes in a new direction. This complex problem is hard to unravel with a single study, especially in the absence of experimentation. Yet, the main two points of this discovery are loud and clear: social behavior observed in nature may be a product of culture, and even the fiercest primates do not forever need to stay this way.

Let us hope this applies to humanity as well.
